# Author Correction: Compartments in medulloblastoma with extensive nodularity are connected through differentiation along the granular precursor lineage

**DOI:** 10.1038/s41467-024-52880-8

**Published:** 2024-10-04

**Authors:** David R. Ghasemi, Konstantin Okonechnikov, Anne Rademacher, Stephan Tirier, Kendra K. Maass, Hanna Schumacher, Piyush Joshi, Maxwell P. Gold, Julia Sundheimer, Britta Statz, Ahmet S. Rifaioglu, Katharina Bauer, Sabrina Schumacher, Michele Bortolomeazzi, Felice Giangaspero, Kati J. Ernst, Steven C. Clifford, Julio Saez-Rodriguez, David T. W. Jones, Daisuke Kawauchi, Ernest Fraenkel, Jan-Philipp Mallm, Karsten Rippe, Andrey Korshunov, Stefan M. Pfister, Kristian W. Pajtler

**Affiliations:** 1 Hopp-Children’s Cancer Center Heidelberg (KiTZ), Heidelberg, Germany; 2 Division of Pediatric Neuro-oncology, German Cancer Consortium (DKTK), German Cancer Research Center (DKFZ), Heidelberg, Germany; 3 Department of Pediatric Oncology, Hematology, and Immunology, Heidelberg University Hospital, Heidelberg, Germany; 4 Division of Chromatin Networks, German Cancer Research Center (DKFZ) and Bioquant, Heidelberg, Germany; 5 Heidelberg University, Faculty of Medicine, and Heidelberg University Hospital, Institute for Computational Biomedicine, Bioquant, Heidelberg, Germany; 6 Department of Biological Engineering, Massachusetts Institute of Technology (MIT), Cambridge, MA USA; 7 Faculty of Biosciences, Heidelberg University, Heidelberg, Germany; 8 Department of Electrical and Electronics Engineering, İskenderun Technical University, Hatay, Turkey; 9 Single-cell Open Lab, German Cancer Research Center (DKFZ), Heidelberg, Germany; 10 Department of Radiological, Oncological and Anatomo-Pathological Sciences, Sapienza University of Rome, Rome, Italy; 11 Istituto di Ricovero e Cura a Carattere Scientifico (IRCCS) Neuromed, Pozzilli, Italy; 12 Division of Pediatric Glioma Research, German Cancer Research Center (DKFZ), Heidelberg, Germany; 13 Wolfson Childhood Cancer Research Centre, Translational and Clinical Research Institute, Newcastle University Centre for Cancer, Newcastle upon Tyne, UK; 14 Department of Biochemistry and Cellular Biology, National Center of Neurology and Psychiatry (NCNP), Tokyo, Japan; 15 Edythe Broad Institute of MIT and Harvard, Cambridge, MA USA; 16 Department of Neuropathology, Institute of Pathology, Heidelberg University Hospital, Heidelberg, Germany; 17 Clinical Cooperation Unit Neuropathology, German Cancer Research Center (DKFZ), German Consortium for Translational Cancer Research (DKTK), Heidelberg, Germany; 18 Present Address: Resolve BioSciences GmbH, Monheim am Rhein, Germany

**Keywords:** CNS cancer, Paediatric cancer, Tumour heterogeneity, Differentiation, Oncogenesis

Correction to: *Nature Communications* 10.1038/s41467-023-44117-x, published online 08 January 2024

In the Acknowledgements section, the following sentence ‘The development of spatial transcriptomics protocol was supported by the START-HD project within the HMLS Explorer program of the University of Heidelberg’ should have read ‘The development of spatial transcriptomics protocols was supported by the Baden- Württemberg Stiftung (project MET-ID41-STARFISH) and by the HMLS Explorer program of the University of Heidelberg (project START-HD). The original article has been corrected.

