# Supplementary material for: Compartments in medulloblastoma with extensive nodularity are connected through differentiation along the granular precursor lineage
Source: Nat Commun. 2024 Jan 8;15:269. doi: 10.1038/s41467-023-44117-x (PMC10774372; doi:10.1038/s41467-023-44117-x)
Supplement: Supplementary file 1 — Supplementary Information [file 41467_2023_44117_MOESM1_ESM.pdf]

Supplementary Figures

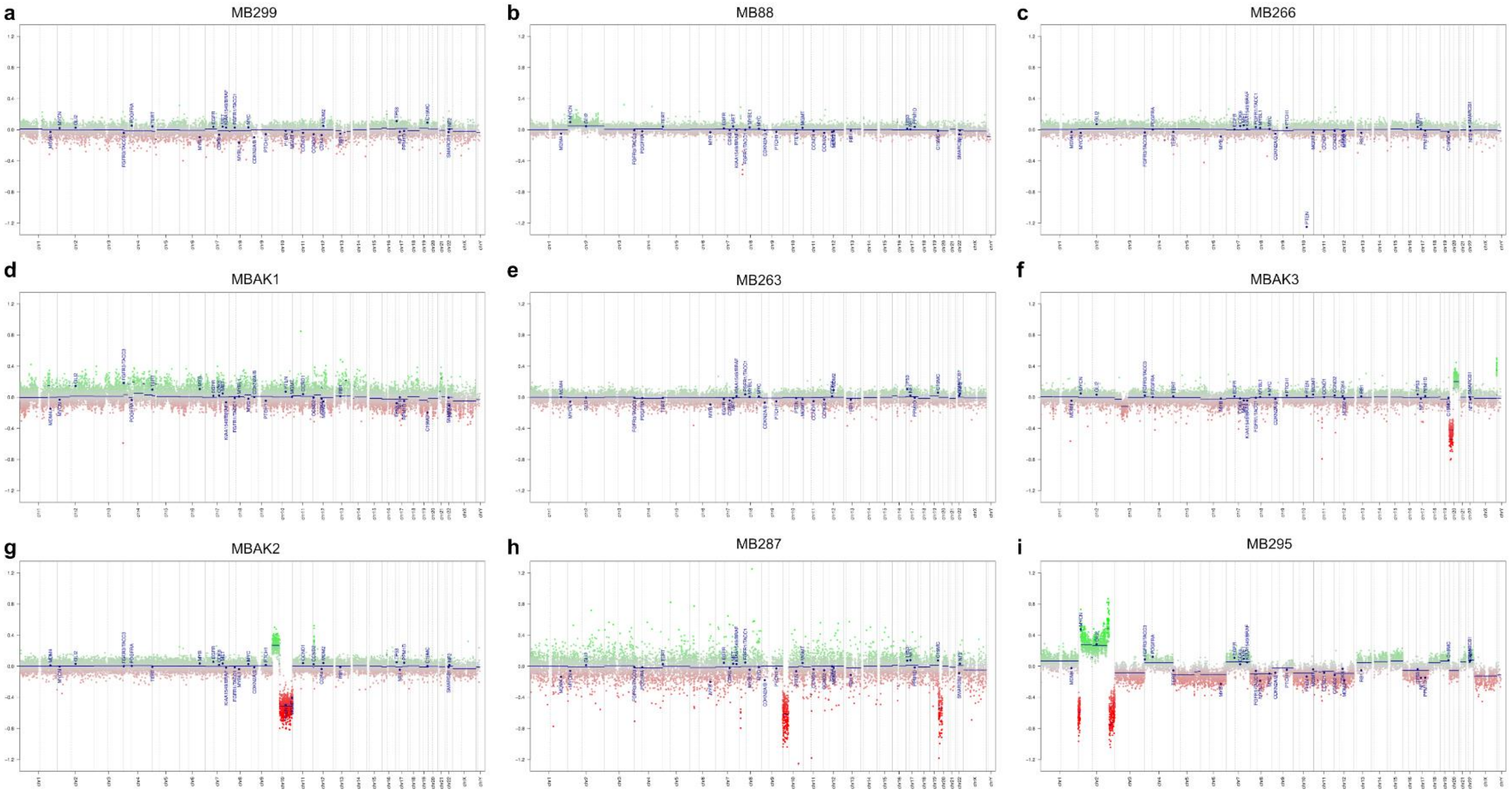

Suppl. Fig. 1: CNVs are infrequent in MBEN.

a – i CNV-plots derived from DNA-methylation profiling data for all nine samples (no chromosomal CNAs: five cases; 1-2 chromosomal CNAs: three cases; >2 chromosomal CNAs: one case).

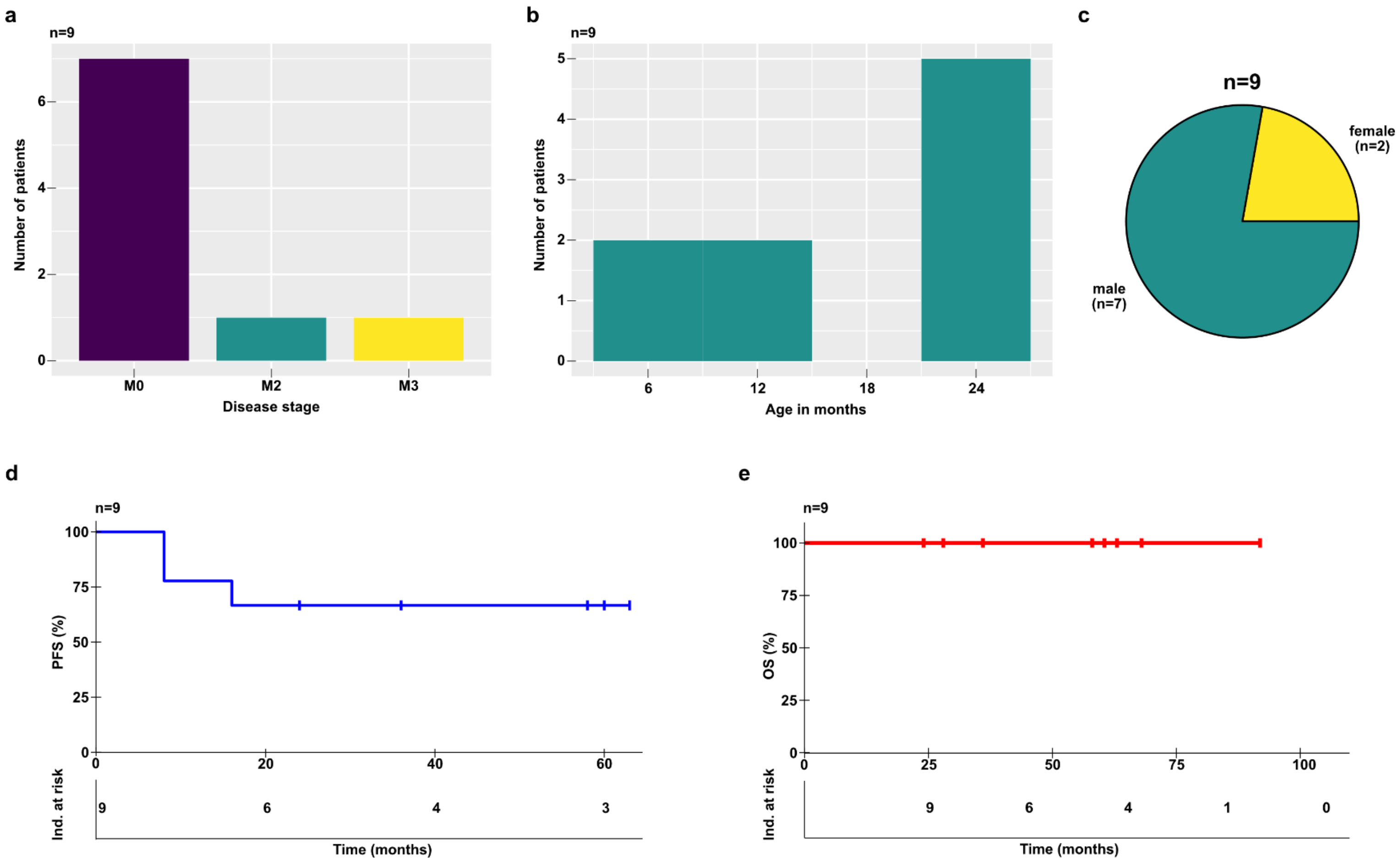

Suppl. Fig. 2: Clinical and epidemiological features of the MBEN patient cohort.

a Bar chart depicting disease stage at time of diagnosis **b** Histogram of age in years at diagnosis **c** Pie chart of the sex distribution of the cohort **d** Kaplan-Meier-curve showing PFS **e** Kaplan-Meier-curve showing OS. PFS = progression free survival, OS = overall survival. All panels: n = 9. Source data are provided as a Source Data file.

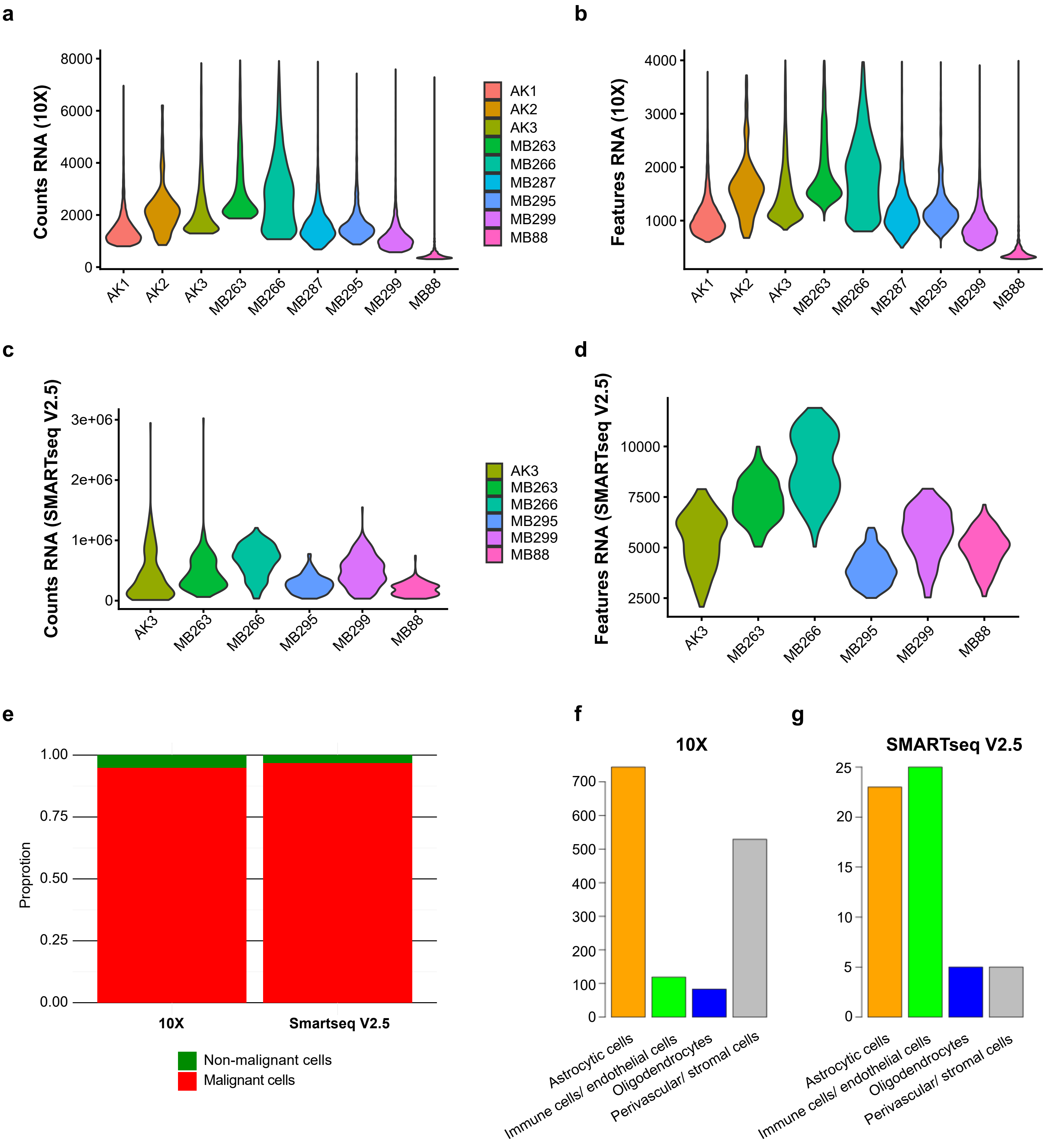
a
Counts RNA (10X)
8000
6000
4000
2000
0
AK1
AK2
AK3
MB263
MB266
MB287
MB295
MB299
MB88
b
Features RNA (10X)
4000
3000
2000
1000
c
Counts RNA (SMARTseq V2.5)
3e+06
2e+06
1e+06
0
d
Features RNA (SMARTseq V2.5)
10000
7500
5000
2500
e
Proprotion
1.00
0.75
0.50
0.25
0.00
10X
Smartseq V2.5
Non-malignant cells
Malignant cells
f
10X
700
600
500
400
300
200
100
0
Astrocytic cells
Immune cells/ endothelial cells
Oligodendrocytes
Perivascular/ stromal cells
g
SMARTseq V2.5
25
20
15
10
5
0

Suppl. Fig. 3: Quality control and comparability of snRNA-seq using 10X Genomics 3'-V2 and SMARTseq V2.5.

a RNA counts per cell for each patient as detected with 10 Genomics 3'-V2 snRNA-seq. **b** Feature counts per cell for each patient as detected with 10 Genomics 3'-V2 snRNA-seq. **c** RNA counts per cell for each patient as detected with SMARTseq V2.5 snRNA-seq. **d** Feature counts per cell for each patient as detected with SMARTseq V2.5 snRNA-seq. **e** Stacked bar chart showing the amount of non-malignant and malignant cells for both methods. **f** Bar chart showing the different non-malignant cells that were identified using the 10X protocol. **g** Bar chart showing the different non-malignant cells that were identified using the SMARTseq V2.5 protocol. Source data are provided as a Source Data file.

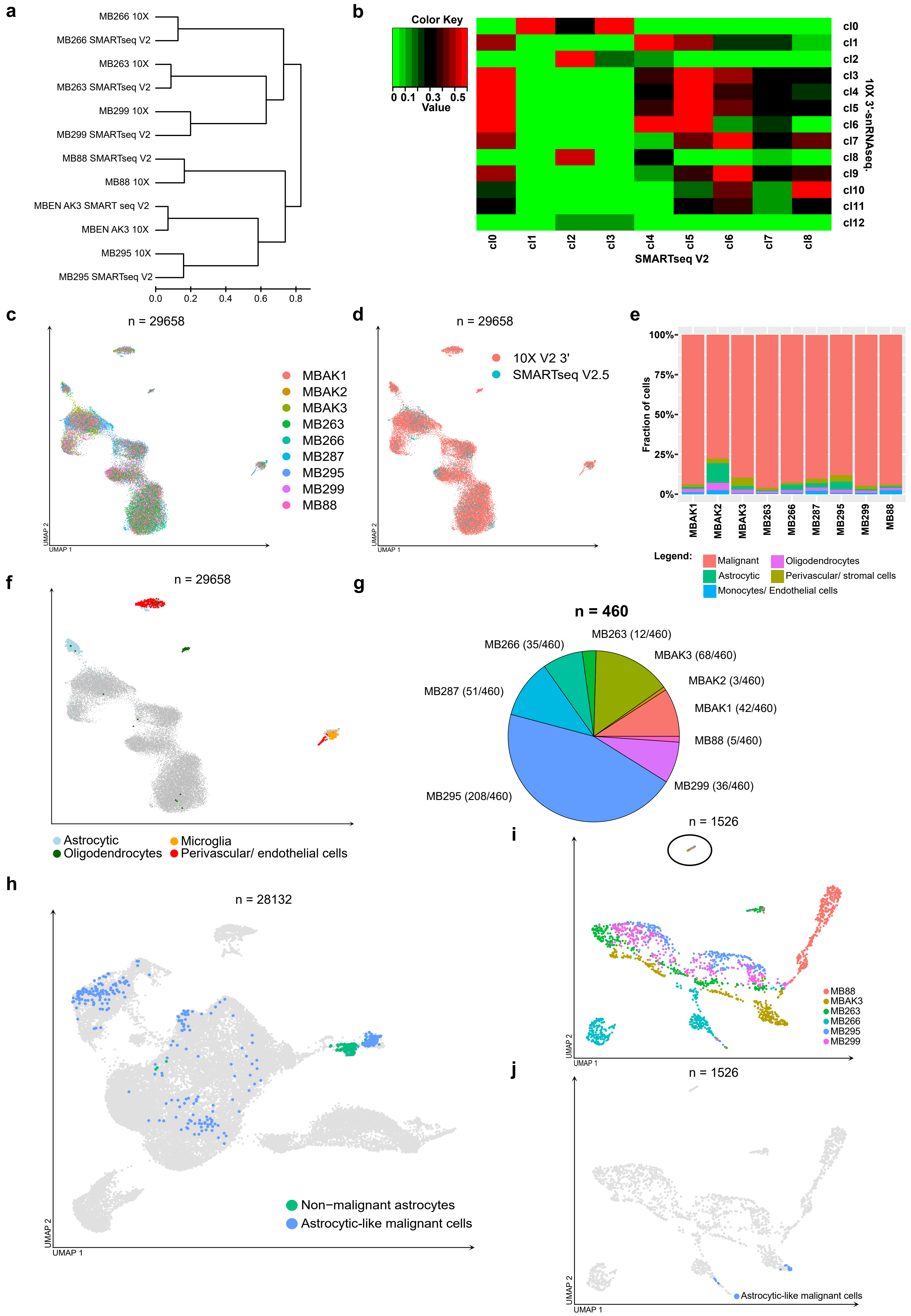

a
MB266 10X
MB266 SMARTseq V2
MB263 10X
MB263 SMARTseq V2
MB299 10X
MB299 SMARTseq V2
MB88 SMARTseq V2
MB88 10X
MBEN AK3 SMART seq V2
MBEN AK3 10X
MB295 10X
MB295 SMARTseq V2
0.0 0.2 0.4 0.6 0.8
b
Color Key
0 0.1 0.3 0.5
Value
cl0 cl1 cl2 cl3 cl4 cl5 cl6 cl7 cl8 cl9 cl10 cl11 cl12
10X 3'-snRNAseq.
cl0 cl1 cl2 cl3 cl4 cl5 cl6 cl7 cl8
SMARTseq V2
c
n = 29658
MBAK1
MBAK2
MBAK3
MB263
MB266
MB287
MB295
MB299
MB88
UMAP 1
UMAP 2
d
n = 29658
10X V2 3'
SMARTseq V2.5
UMAP 1
UMAP 2
e
100%
75%
50%
25%
0%
Fraction of cells
MBAK1 MBAK2 MBAK3 MB263 MB266 MB287 MB295 MB299 MB88
Legend:
Malignant
Oligodendrocytes
Astrocytic
Perivascular/ stromal cells
Monocytes/ Endothelial cells
f
n = 29658
Astrocytic
Microglia
Oligodendrocytes
Perivascular/ endothelial cells
g
n = 460
MB263 (12/460)
MB266 (35/460)
MBAK3 (68/460)
MBAK2 (3/460)
MB287 (51/460)
MBAK1 (42/460)
MB88 (5/460)
MB299 (36/460)
MB295 (208/460)
h
n = 28132
Non−malignant astrocytes
Astrocytic-like malignant cells
UMAP 1
UMAP 2
i
n = 1526
MB88
MBAK3
MB263
MB266
MB295
MB299
UMAP 1
UMAP 2
j
n = 1526
Astrocytic-like malignant cells
UMAP 1
UMAP 2

Suppl. Fig. 4: Batch effect control and identification of non-malignant cell types as well as astrocytic-like malignant cells based on snRNA-seq derived CNV-analysis.

a Unsupervised clustering of 10X and SMARTseq V2 genotype profiles confirms strong transcriptional similarity between clusters derived from both snRNA-seq methods (n = 6). **b** Heatmap showing clear correlation between clusters of single cells sequenced with two complementary methods (x-axis: SMARTseq. V2, y-axis: 10X Genomics). **c** UMAP-projection colorized according to patient. No patient-specific batch effects were detectable after integration (n = 9 patients). **d** UMAP-projection colorized according to technology confirms good integration across both technological platforms. **e** Stacked bar chart visualizing the distribution of cells across malignant and non-malignant clusters for every patient. **f** Non-malignant cells were mapped onto an atlas of the developing cerebellum to confirm the designation of non-malignant cell types (monocytes/microglia, astrocytic cells, oligodendrocytes, perivascular and endothelial). **g** Pie chart summarizing the number of astrocytic-like malignant cells per sample. All samples are represented in varying degrees. **h** UMAP-projection of the 10X snRNA-seq dataset without correction for batch effects (also compare: Fig. 3g). Cells from the astrocytic cluster in (f) are highlighted. Whereas one part of the cells with an astrocytic phenotype (blue) cluster with malignant cells, the other part falls into one of the two mixed cluster of non-malignant cells (green) (n = 9 patients). **i** UMAP projection of the SMARTseq V2.5 snRNA-seq. dataset which was not corrected for patient batch effect. Whereas the majority of cells cluster according to patient, significant mixing of cells occurs in one cluster (encircled), representing non-malignant monocytic cells (n = 6 patients). **j** UMAP projection (SMARTseq V2.5 dataset) in which astrocytic-like cells are highlighted. No cells from of the astrocytic cluster (compare f) falls into the mixed, non-malignant cluster (n = 6 patients). Source data are provided as a Source Data file.

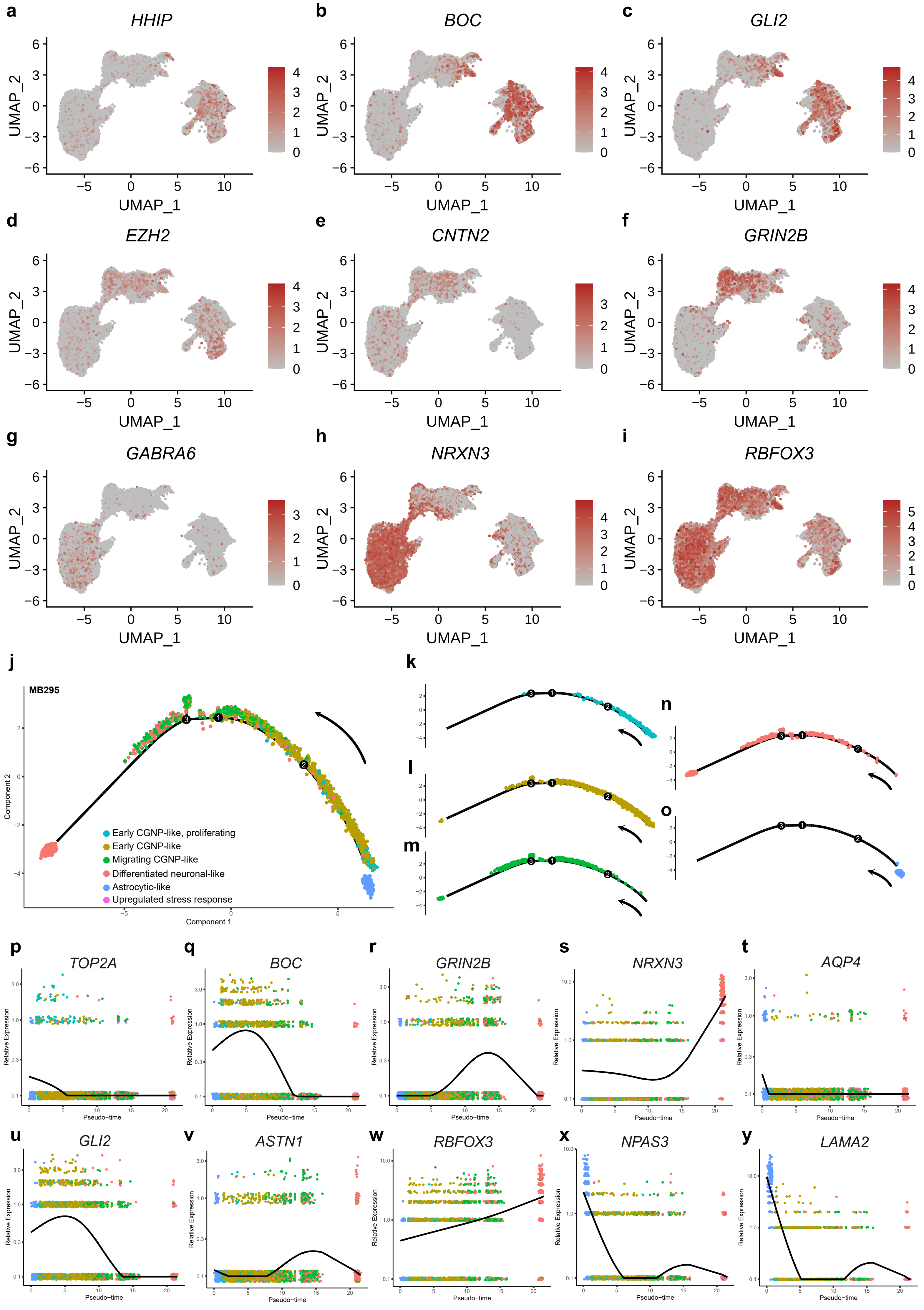
a
HHIP
b
BOC
c
GLI2
d
EZH2
e
CNTN2
f
GRIN2B
g
GABRA6
h
NRXN3
i
RBFOX3
UMAP_1
UMAP_2
j
MB295
Early CGNP-like, proliferating
Early CGNP-like
Migrating CGNP-like
Differentiated neuronal-like
Astrocytic-like
Upregulated stress response
Component 1
Component 2
k
l
m
n
o
p
TOP2A
q
BOC
r
GRIN2B
s
NRXN3
t
AQP4
u
GLI2
v
ASTN1
w
RBFOX3
x
NPAS3
y
LAMA2
Relative Expression
Pseudo−time

Suppl. Fig. 5: Malignant cell states differ in their expression of CGNP-marker genes.

a – i UMAP-plots showing the expression of representative marker genes of CGNP development. **a – c** SHH-pathway members *HHIP, BOC,* and *GLI2*. **d** epigenetic regulator gene *EZH2.* **e, f** markers of intermediate CGNP-stages *CNTN2* and *GRIN2B*. **g – i** markers of differentiated CGNPs *GABRA6*, *NRXN3*, and *RBFOX3*. **j** Cell fate trajectory of the sample MB295 as constructed with monocle2. The black arrow shows the direction of the trajectory. **k – o** Projections showing the position of the different cell stages on the MBEN trajectory in sample MB295. The color code is equivalent to (j). **p – y** Visualizations of the relative expression of different marker genes along the MBEN trajectory in MB295. All single images relate to n = 9 patients / n = 27782 cells each. Source data are provided as a Source Data file.

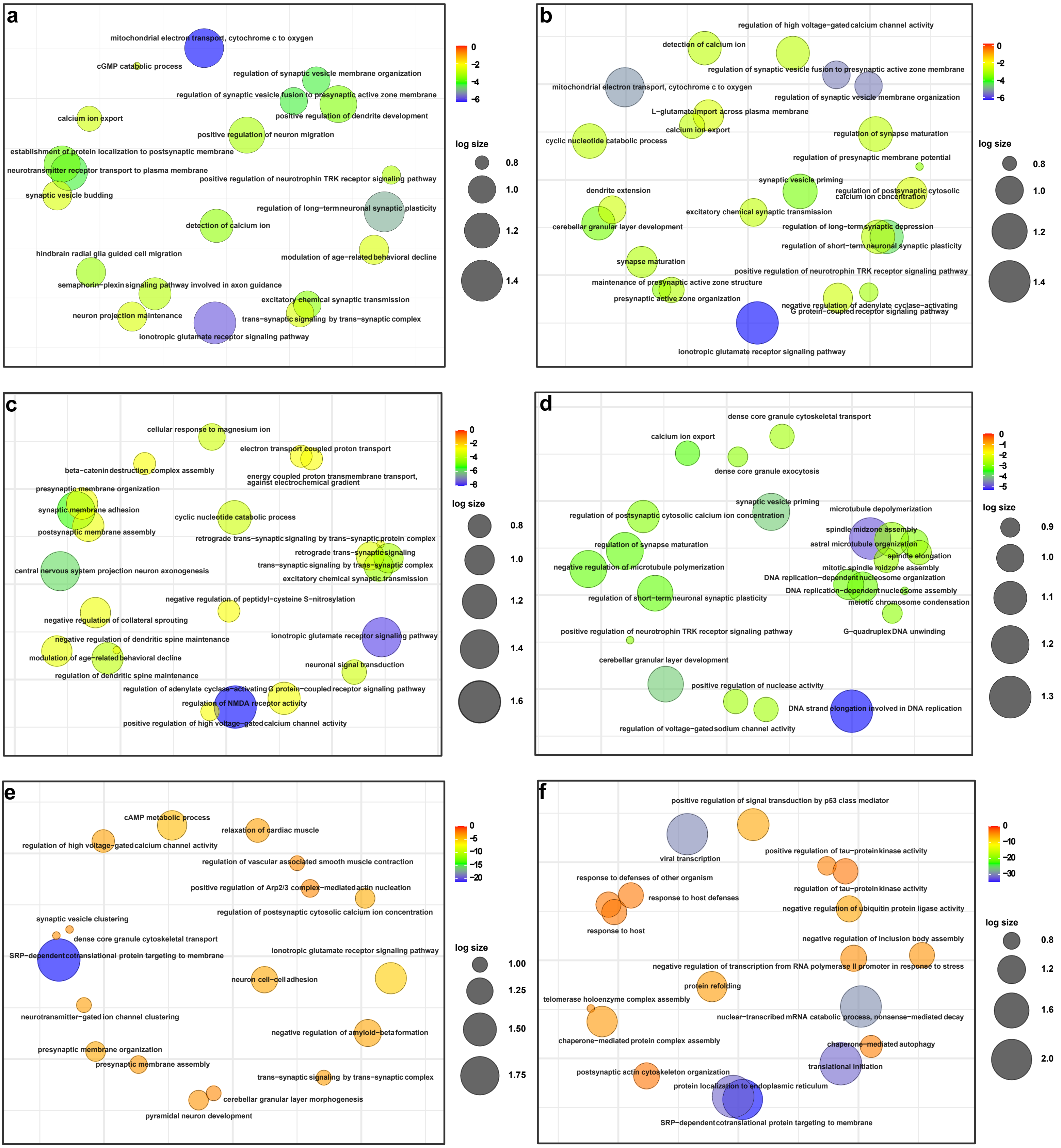
a
mitochondrial electron transport, cytochrome c to oxygen
cGMP catabolic process
regulation of synaptic vesicle membrane organization
regulation of synaptic vesicle fusion to presynaptic active zone membrane
calcium ion export
positive regulation of dendrite development
positive regulation of neuron migration
establishment of protein localization to postsynaptic membrane
neurotransmitter receptor transport to plasma membrane
positive regulation of neurotrophin TRK receptor signaling pathway
synaptic vesicle budding
regulation of long−term neuronal synaptic plasticity
detection of calcium ion
hindbrain radial glia guided cell migration
modulation of age−related behavioral decline
semaphorin−plexin signaling pathway involved in axon guidance
excitatory chemical synaptic transmission
neuron projection maintenance
trans−synaptic signaling by trans−synaptic complex
ionotropic glutamate receptor signaling pathway
0
−2
−4
−6
log size
0.8
1.0
1.2
1.4
b
regulation of high voltage−gated calcium channel activity
detection of calcium ion
regulation of synaptic vesicle fusion to presynaptic active zone membrane
mitochondrial electron transport, cytochrome c to oxygen
regulation of synaptic vesicle membrane organization
L−glutamate import across plasma membrane
calcium ion export
regulation of synapse maturation
cyclic nucleotide catabolic process
regulation of presynaptic membrane potential
synaptic vesicle priming
dendrite extension
regulation of postsynaptic cytosolic calcium ion concentration
excitatory chemical synaptic transmission
cerebellar granular layer development
regulation of long−term synaptic depression
regulation of short−term neuronal synaptic plasticity
synapse maturation
positive regulation of neurotrophin TRK receptor signaling pathway
maintenance of presynaptic active zone structure
presynaptic active zone organization
negative regulation of adenylate cyclase−activating G protein−coupled receptor signaling pathway
ionotropic glutamate receptor signaling pathway
0
−2
−4
−6
log size
0.8
1.0
1.2
1.4
c
cellular response to magnesium ion
electron transport coupled proton transport
beta−catenin destruction complex assembly
energy coupled proton transmembrane transport, against electrochemical gradient
presynaptic membrane organization
synaptic membrane adhesion
cyclic nucleotide catabolic process
postsynaptic membrane assembly
retrograde trans−synaptic signaling by trans−synaptic protein complex
retrograde trans−synaptic signaling
trans−synaptic signaling by trans−synaptic complex
central nervous system projection neuron axonogenesis
excitatory chemical synaptic transmission
negative regulation of peptidyl−cysteine S−nitrosylation
negative regulation of collateral sprouting
negative regulation of dendritic spine maintenance
ionotropic glutamate receptor signaling pathway
modulation of age−related behavioral decline
neuronal signal transduction
regulation of dendritic spine maintenance
regulation of adenylate cyclase−activating G protein−coupled receptor signaling pathway
regulation of NMDA receptor activity
positive regulation of high voltage−gated calcium channel activity
0
−2
−4
−6
−8
log size
0.8
1.0
1.2
1.4
1.6
d
dense core granule cytoskeletal transport
calcium ion export
dense core granule exocytosis
synaptic vesicle priming
regulation of postsynaptic cytosolic calcium ion concentration
microtubule depolymerization
spindle midzone assembly
regulation of synapse maturation
astral microtubule organization
spindle elongation
negative regulation of microtubule polymerization
mitotic spindle midzone assembly
DNA replication−dependent nucleosome organization
DNA replication−dependent nucleosome assembly
regulation of short−term neuronal synaptic plasticity
meiotic chromosome condensation
positive regulation of neurotrophin TRK receptor signaling pathway
G−quadruplex DNA unwinding
cerebellar granular layer development
positive regulation of nuclease activity
DNA strand elongation involved in DNA replication
regulation of voltage−gated sodium channel activity
0
−1
−2
−3
−4
−5
log size
0.9
1.0
1.1
1.2
1.3
e
cAMP metabolic process
relaxation of cardiac muscle
regulation of high voltage−gated calcium channel activity
regulation of vascular associated smooth muscle contraction
positive regulation of Arp2/3 complex−mediated actin nucleation
regulation of postsynaptic cytosolic calcium ion concentration
synaptic vesicle clustering
dense core granule cytoskeletal transport
SRP−dependent cotranslational protein targeting to membrane
ionotropic glutamate receptor signaling pathway
neuron cell−cell adhesion
neurotransmitter−gated ion channel clustering
negative regulation of amyloid−beta formation
presynaptic membrane organization
presynaptic membrane assembly
trans−synaptic signaling by trans−synaptic complex
cerebellar granular layer morphogenesis
pyramidal neuron development
0
−5
−10
−15
−20
log size
1.00
1.25
1.50
1.75
f
positive regulation of signal transduction by p53 class mediator
viral transcription
positive regulation of tau−protein kinase activity
response to defenses of other organism
regulation of tau−protein kinase activity
response to host defenses
negative regulation of ubiquitin protein ligase activity
response to host
negative regulation of inclusion body assembly
negative regulation of transcription from RNA polymerase II promoter in response to stress
protein refolding
telomerase holoenzyme complex assembly
nuclear−transcribed mRNA catabolic process, nonsense−mediated decay
chaperone−mediated protein complex assembly
chaperone−mediated autophagy
translational initiation
postsynaptic actin cytoskeleton organization
protein localization to endoplasmic reticulum
SRP−dependent cotranslational protein targeting to membrane
0
−10
−20
−30
log size
0.8
1.2
1.6
2.0

Suppl. Fig. 6: Gene ontology analysis reveals core biological functions of granular cerebellar development to be upregulated in MBEN.

Clustering visualizations based on semantic similarity representing the top 25 GO terms based on fold enrichment for **a** differentiated, neuronal-like, **b** early CGNP-like, **c** migrating CGNP-like, **d** early CGNP-like, proliferating, **e** astrocytic-like cells, and **f** upregulated stress response. Colorization is based on raw p-values, which were calculated using one-sided Fisher's Exact Tests (PANTHER Overrepresentation Test (Released 20210224)). Source data are provided as a Source Data file.

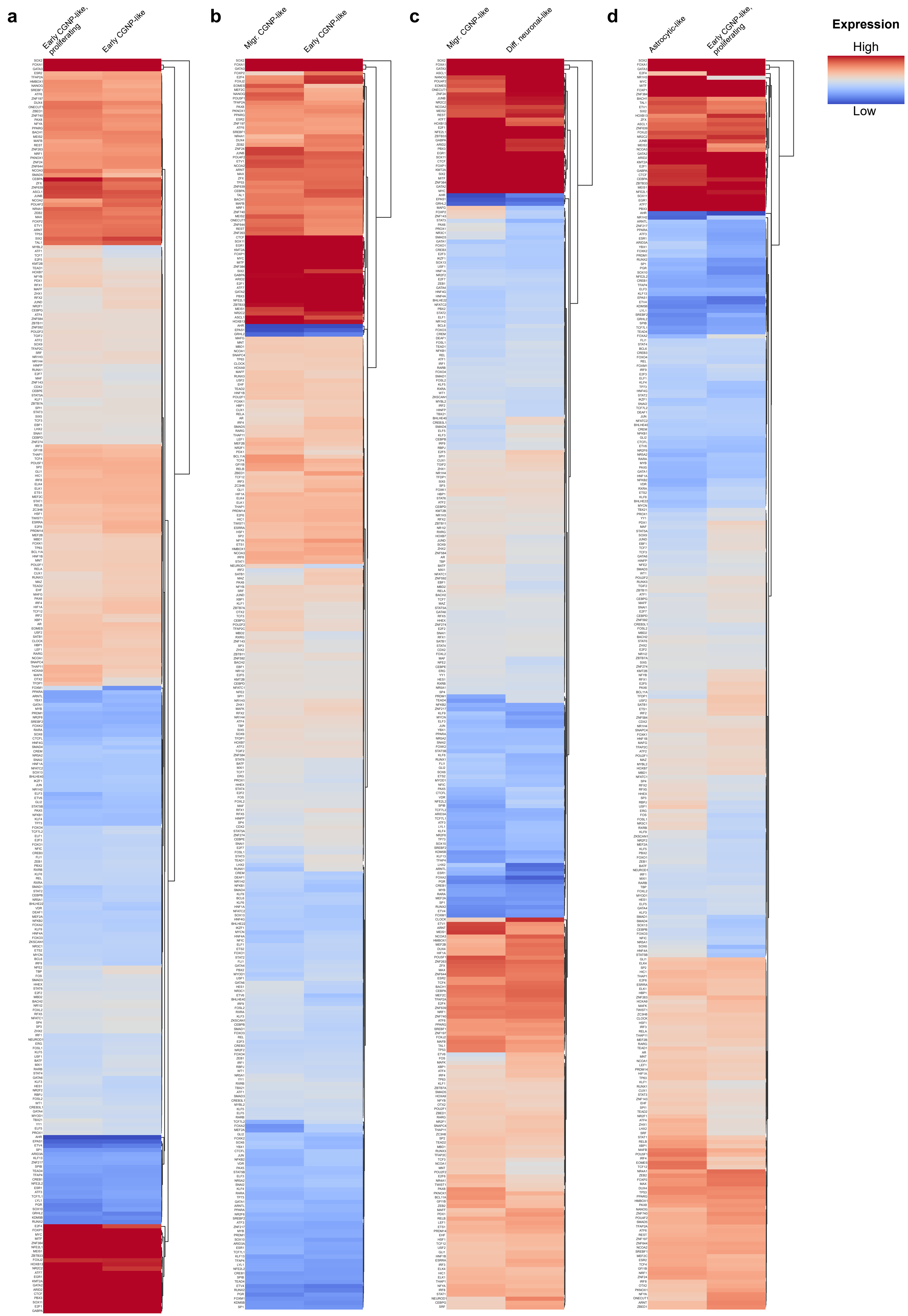
a
Early CGNP-like, proliferating
Early CGNP-like
b
Migr. CGNP-like
Early CGNP-like
c
Migr. CGNP-like
Diff. neuronal-like
d
Astrocytic-like
Early CGNP-like, proliferating
Expression
High
Low

Suppl. Fig. 7: Transcription factor activity changes throughout the course of MBEN development.

a – d Heatmaps visualizing the changes in TF activity during MBEN differentiation. Each heatmap shows the differential TF activity between two clusters (also compare Fig. 3d and 4e and Supplementary Data 5). Source data are provided as a Source Data file.

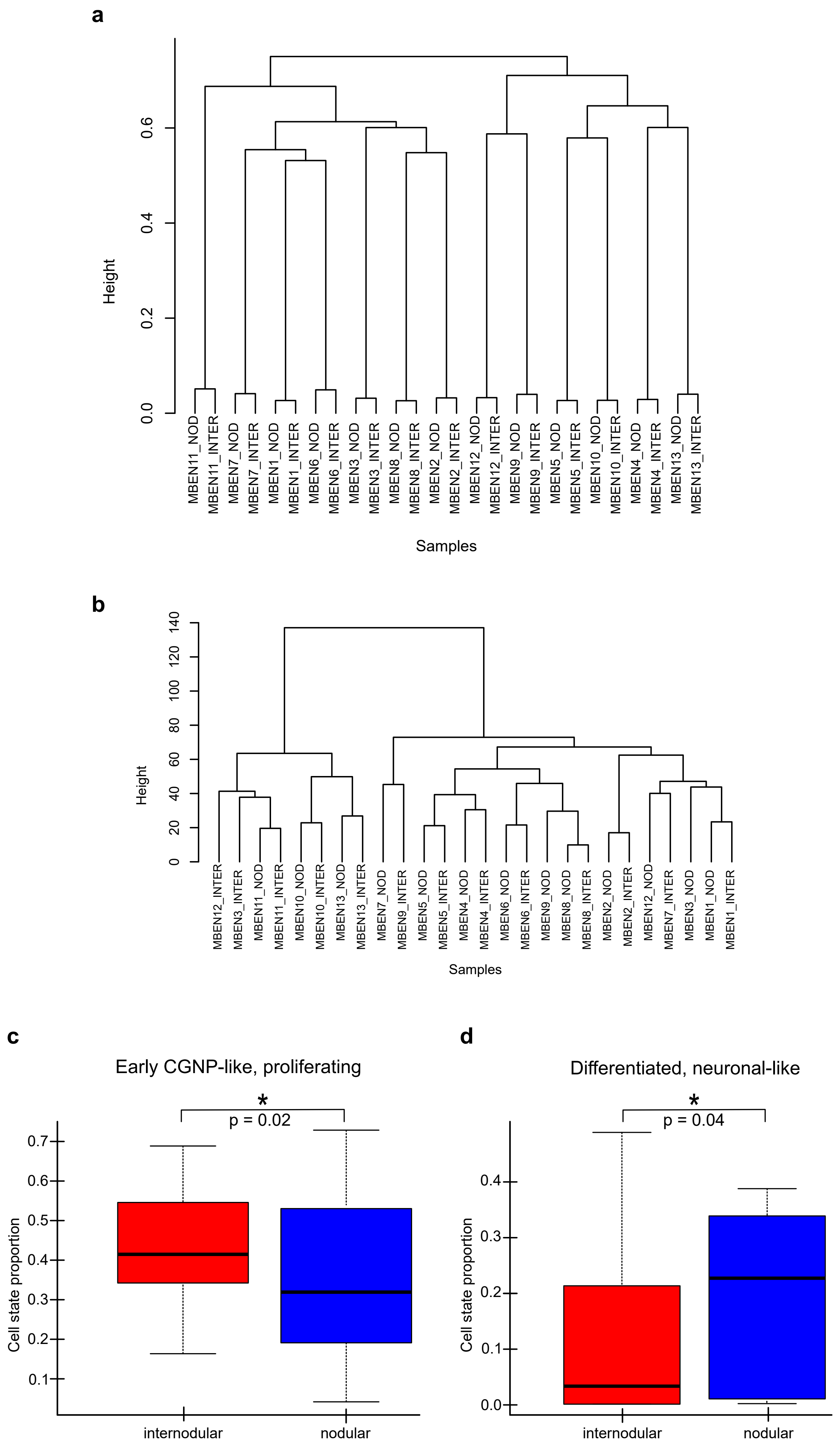
a
Height
0.0
0.2
0.4
0.6
MBEN11_NOD
MBEN11_INTER
MBEN7_NOD
MBEN7_INTER
MBEN1_NOD
MBEN1_INTER
MBEN6_NOD
MBEN6_INTER
MBEN3_NOD
MBEN3_INTER
MBEN8_NOD
MBEN8_INTER
MBEN2_NOD
MBEN2_INTER
MBEN12_NOD
MBEN12_INTER
MBEN9_NOD
MBEN9_INTER
MBEN5_NOD
MBEN5_INTER
MBEN10_NOD
MBEN10_INTER
MBEN4_NOD
MBEN4_INTER
MBEN13_NOD
MBEN13_INTER
Samples
b
Height
0
20
40
60
80
100
120
140
MBEN12_INTER
MBEN3_INTER
MBEN11_NOD
MBEN11_INTER
MBEN10_NOD
MBEN10_INTER
MBEN13_NOD
MBEN13_INTER
MBEN7_NOD
MBEN9_INTER
MBEN5_NOD
MBEN5_INTER
MBEN4_NOD
MBEN4_INTER
MBEN6_NOD
MBEN6_INTER
MBEN9_NOD
MBEN8_NOD
MBEN8_INTER
MBEN2_NOD
MBEN2_INTER
MBEN12_NOD
MBEN7_INTER
MBEN3_NOD
MBEN1_NOD
MBEN1_INTER
Samples
c
Early CGNP-like, proliferating
*
p = 0.02
Cell state proportion
0.1
0.2
0.3
0.4
0.5
0.6
0.7
internodular
nodular
d
Differentiated, neuronal-like
*
p = 0.04
Cell state proportion
0.0
0.1
0.2
0.3
0.4
internodular
nodular

Suppl. Fig. 8: Microdissection datasets quality control and inspection.

a Unsupervised hierarchical clustering of internodular and nodular transcriptomic profiles via the top 500 most highly variable genes (applied method: ward.D2) **b** Unsupervised clustering of internodular and nodular genotype profiles confirms tissue source from the same tumor sample. **c, d** Box plots of deconvolution-derived proportions for proliferating, early GCNP-like (c) and differentiated, neuronal like (d) cells as compared between the nodular and internodular compartments (n = 9 patients). * = p-value < 0.05 (two-sided t-Test). The center line, box limits, whiskers, and points indicate the median, upper/lower quartiles, 1.5× interquartile range and outliers, respectively. Source data are provided as a Source Data file.

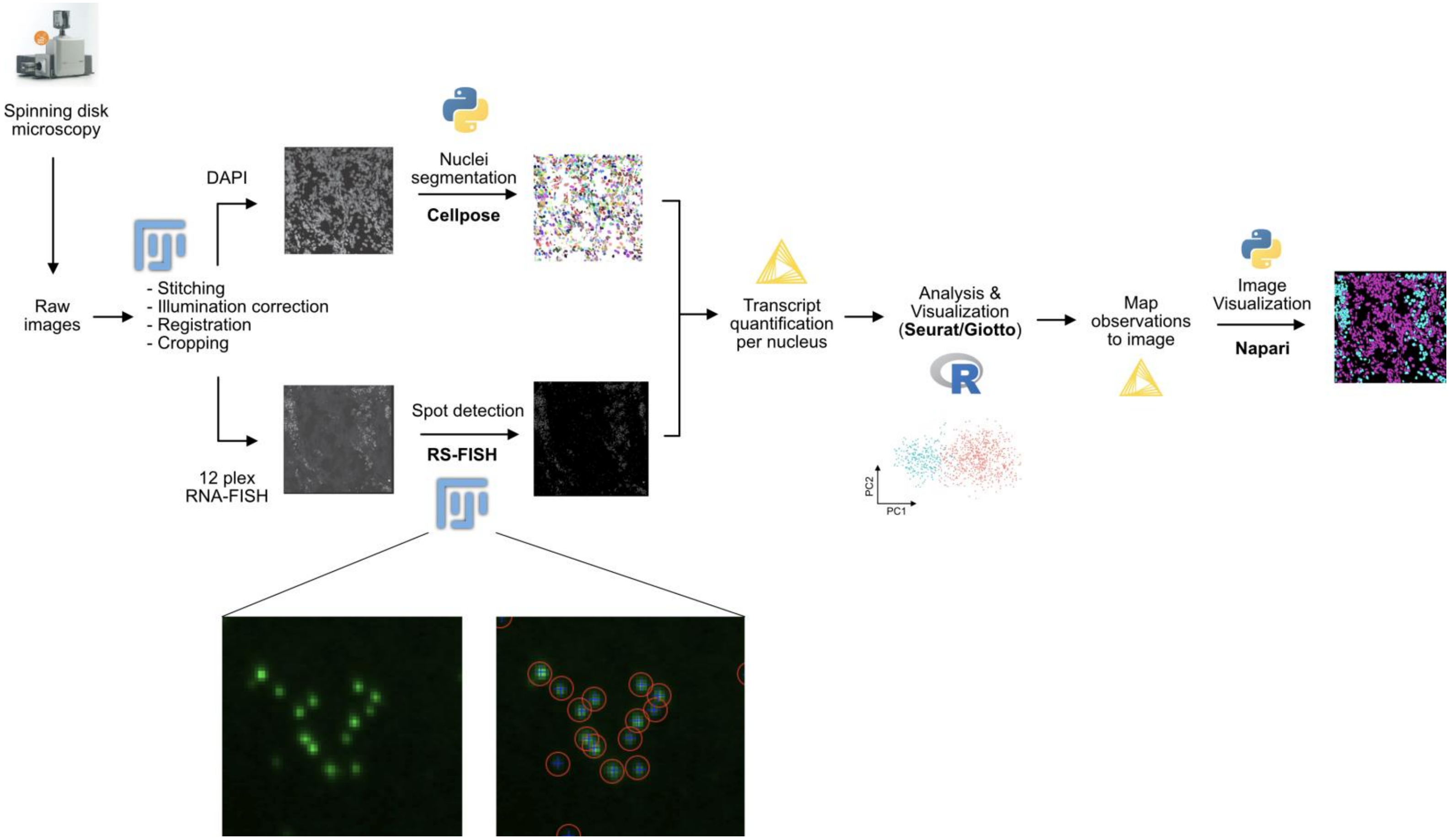

Suppl. Fig. 9: Workflow of the integrated snRNAseq- and RNAscope-analysis.

Workflow depicting the single steps of RNAscope analysis and correlation with clusters from snRNA-seq.

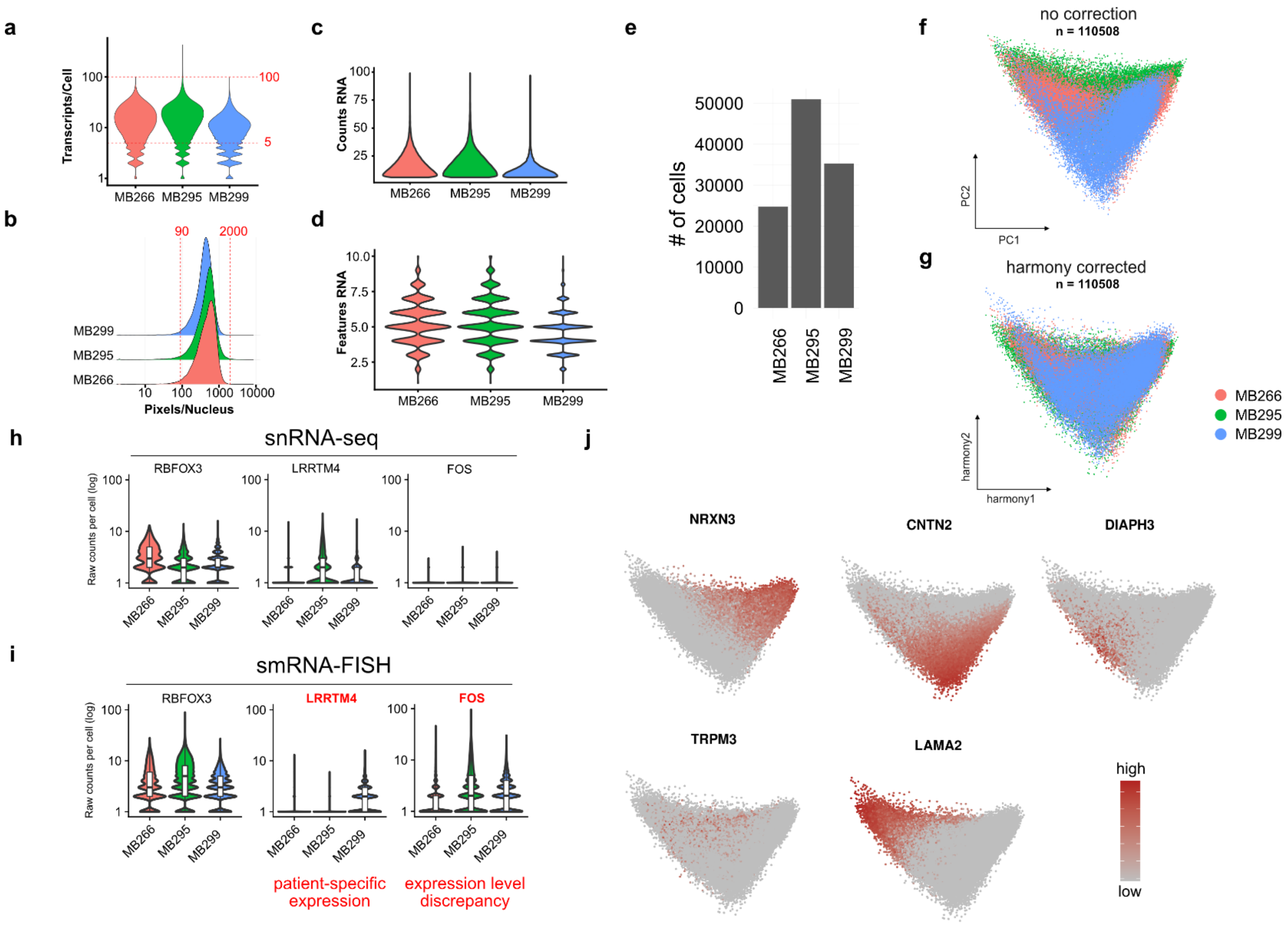

Suppl. Fig. 10: Quality control smRNA-FISH using RNAscope.

a – d Visualizations showing **a** the number of transcripts per single cell, **b** the number of pixels per nucleus, **c** the number of single cells for each sample, and **d** RNA features per cell for each sample. **e** Bar chart showing the number of cells per sample. **f** Principal component analysis showing high concordance between the three cases even without correction, which is further improved by **g** harmony correction. **h, i** Expression of the three marker genes *LRRTM4, FOS*, and *RBFOX3* in the **h** snRNA-seq and **i** smRNA-FISH datasets, respectively. *LRRTM4* and *FOS* were excluded from the further analysis due to strong expression level discrepancies or patient-specific expression (*RBFOX3* shown for comparison). **j** Expression of snRNA-seq derived marker genes in the smRNA-FISH dataset. Source data are provided as a Source Data file.

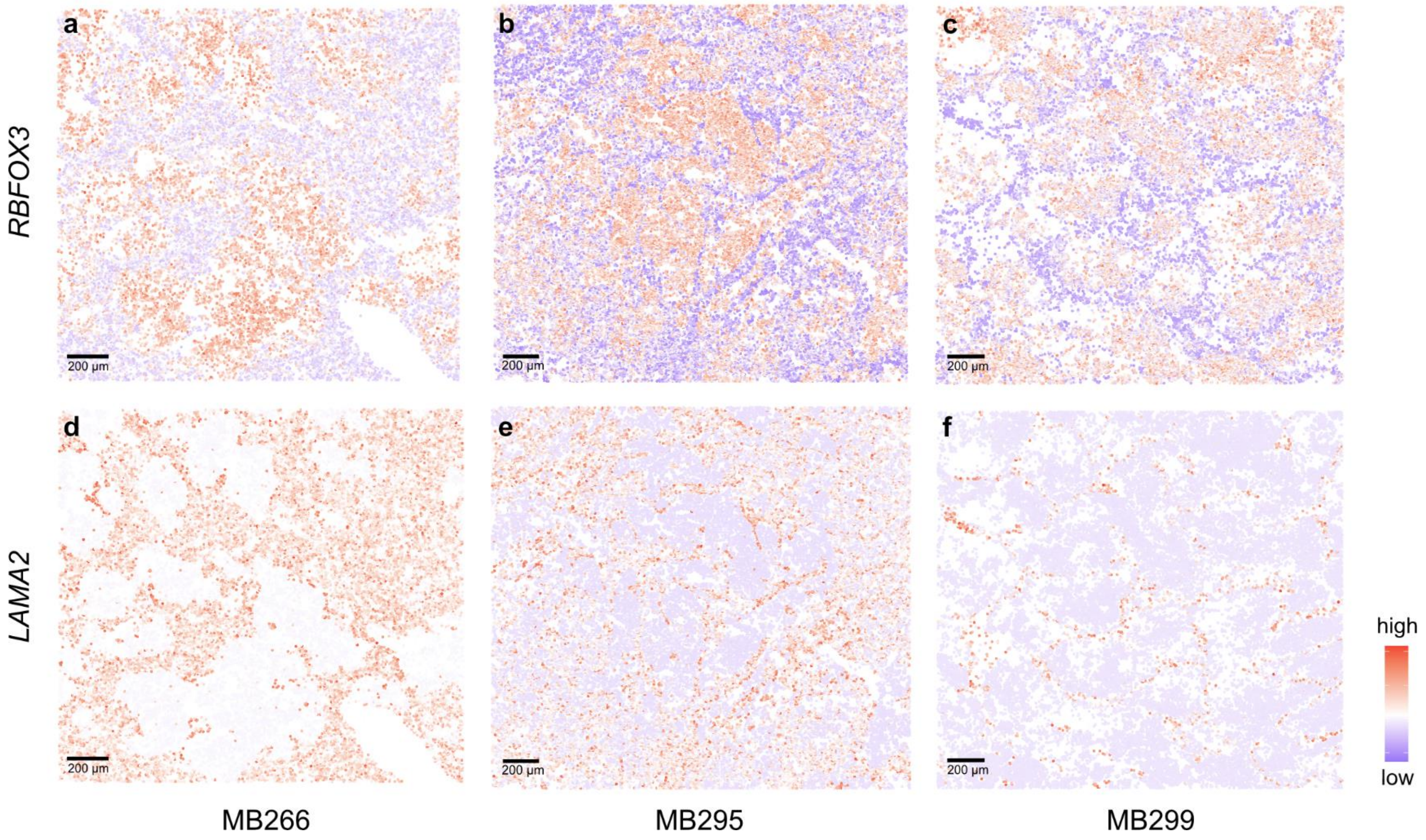

Suppl. Fig. 11: Population-specific marker genes recapitulate MBEN histology.

The marker genes *RBFOX3* and LAMA2 are differentially expressed in all three MBEN samples. **a – c** *RBFOX3* is highly expressed in the nodular compartments, whereas **d - f** *LAMA2*-expression is restricted to the internodular parts of the respective tumors. Size views: MB266: 1924.68 µm x 1931.91 µm, MB295: 2782.7 µm x 2317.81 µm, MB299: 2170.69 µm x 2196.01 µm. Scale bars = 200 µm. Each image is representative of one patient.

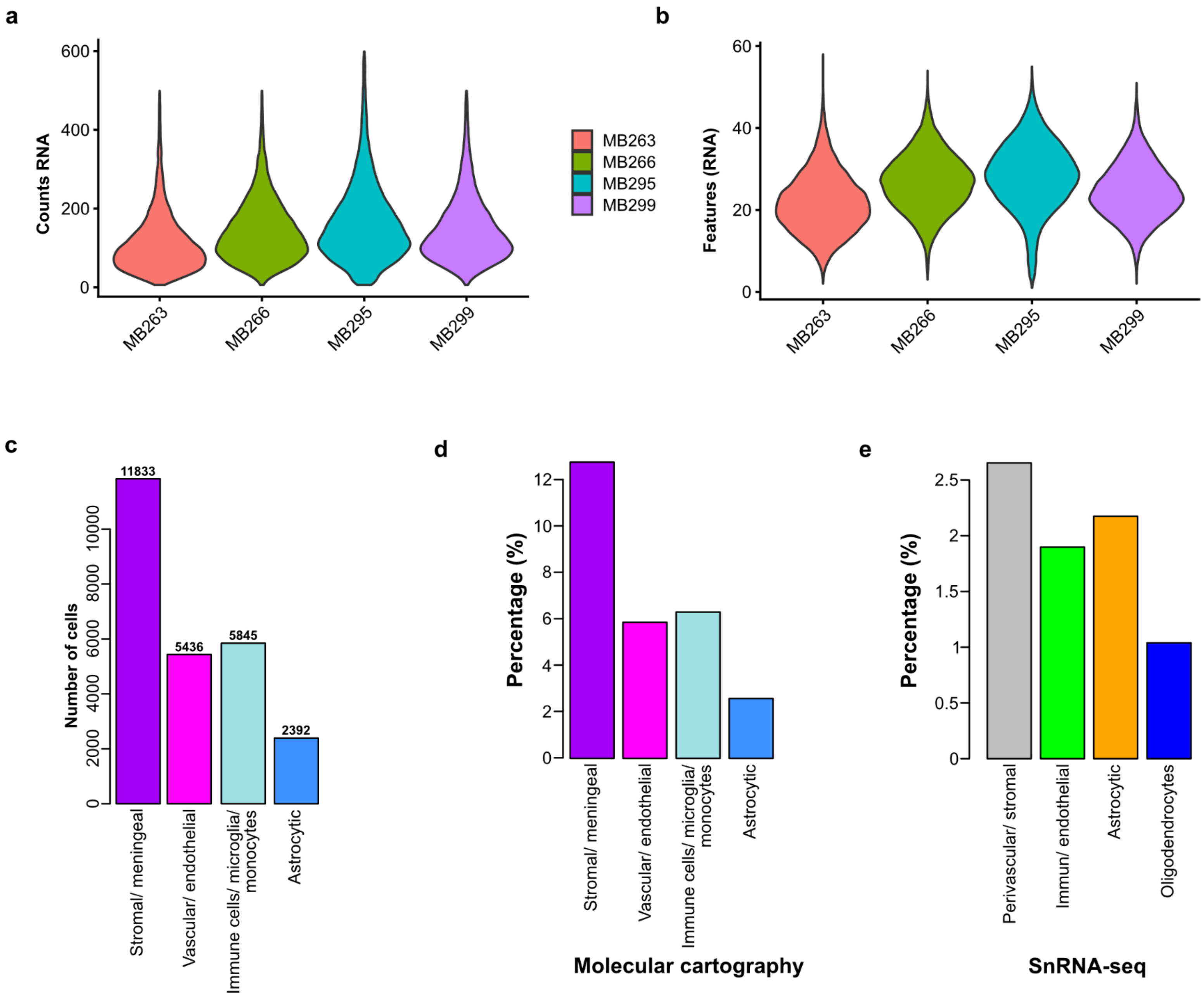

Suppl. Fig. 12: Quality control and comparison of non-malignant cell types between Molecular Cartography and snRNA-seq.

a RNA counts per cell for each sample as detected with Molecular cartography. **b** Number of features per cell for each sample as detected with Molecular cartography. **c** Bar chart summarizing the absolute number of non-malignant and astrocytic-like malignant cells/ astrocytes as detected with Molecular cartography. **d**, **e** Bar charts summarizing the relative abundances of non-malignant cell types and astrocytic-like malignant cells/ astrocytes for (d) Molecular cartography and (e) snRNA-seq. Source data are provided as a Source Data file.

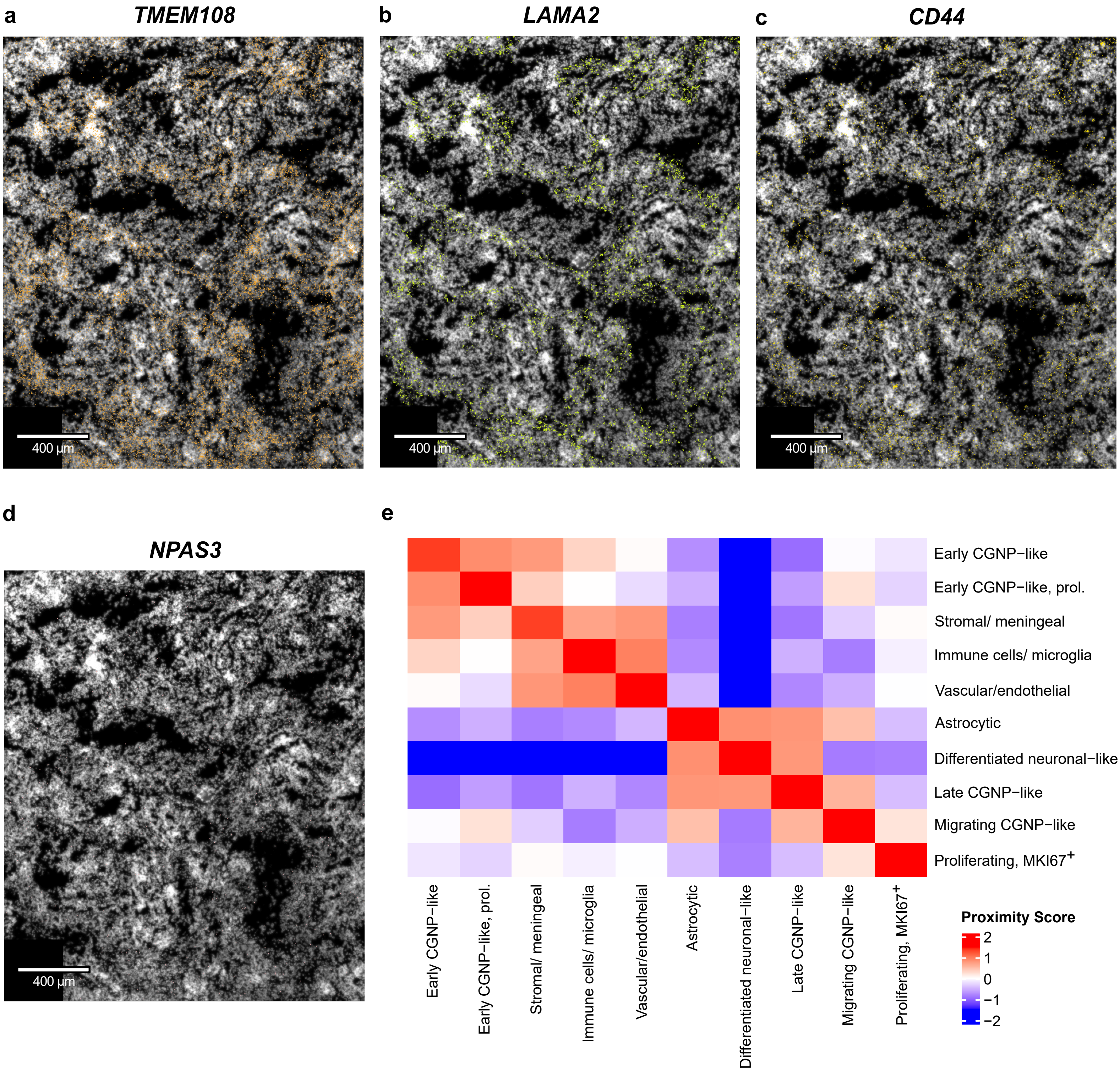
a
TMEM108
400 µm
b
LAMA2
400 µm
c
CD44
400 µm
d
NPAS3
400 µm
e
Early CGNP−like
Early CGNP−like, prol.
Stromal/ meningeal
Immune cells/ microglia
Vascular/endothelial
Astrocytic
Differentiated neuronal−like
Late CGNP−like
Migrating CGNP−like
Proliferating, MKI67+
Proximity Score
2
1
0
−1
−2

Suppl. Fig. 13: The tumor microenvironment in MBEN differs between its two compartments.

Spatial gene expression in sample MB263 of **a** *TMEM108* (marker for the internodular compartment), **b** *LAMA2* (stromal cells, astrocytic cells) **c** *CD44*, and **d** *NPAS3* (both markers for astrocytic cells). **e** Heatmap depicting the integrated cell proximity analysis of all four cases. Cell types which high correlation are more likely to be located next to each other in the tumor microenvironment (based on n = 4 patients). Scale bar in a – d = 400 µm. Source data are provided as a Source Data file.

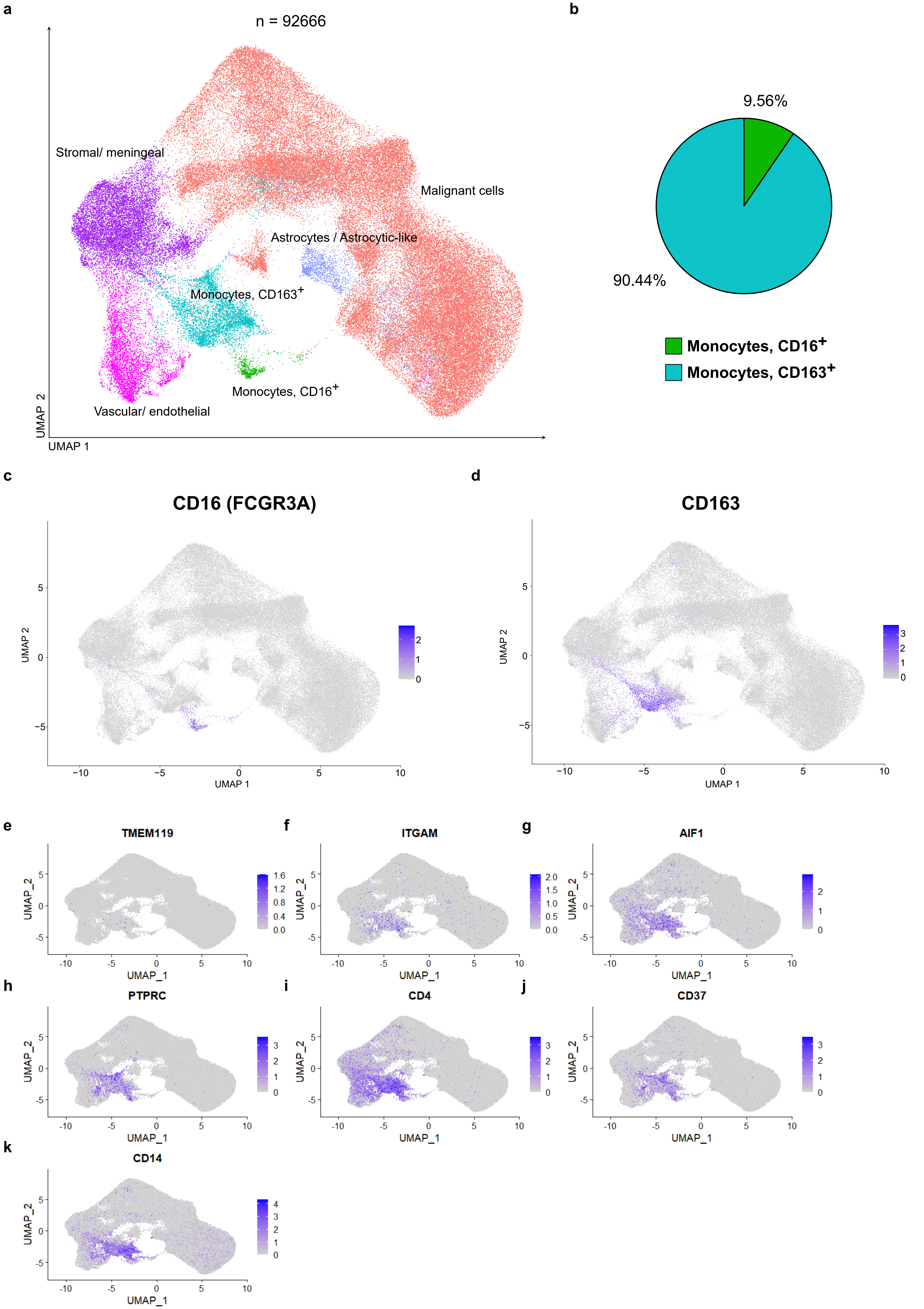
a
n = 92666
Stromal/ meningeal
Malignant cells
Astrocytes / Astrocytic-like
Monocytes, CD163+
Monocytes, CD16+
Vascular/ endothelial
UMAP 2
UMAP 1
b
9.56%
90.44%
Monocytes, CD16+
Monocytes, CD163+
c
CD16 (FCGR3A)
d
CD163
e
TMEM119
f
ITGAM
g
AIF1
h
PTPRC
i
CD4
j
CD37
k
CD14
UMAP_1
UMAP_2

Suppl. Fig. 14: Two distinct types of monocytic cells can be distinguished in the MBEN tumor microenvironment.

a UMAP-projection of single cells derived from molecular cartography. Two distinct types of monocytes can be distinguished based on *CD16*- and *CD163*-expression. **b** pie chart visualizing the proportions of *CD16*- and *CD163*-positive cells, respectively. **c, d** Features plots of (c) *CD16* and (d) *CD163* expression. **e – k** Feature plots showing the expression of the monocytic marker genes *TMEM116, ITGAM, AIF1, PTPRC, CD4, CD37,* and *CD14*. All UMAP-plots relate to n = 92666 cells from n = 4 patients. Source data are provided as a Source Data file.

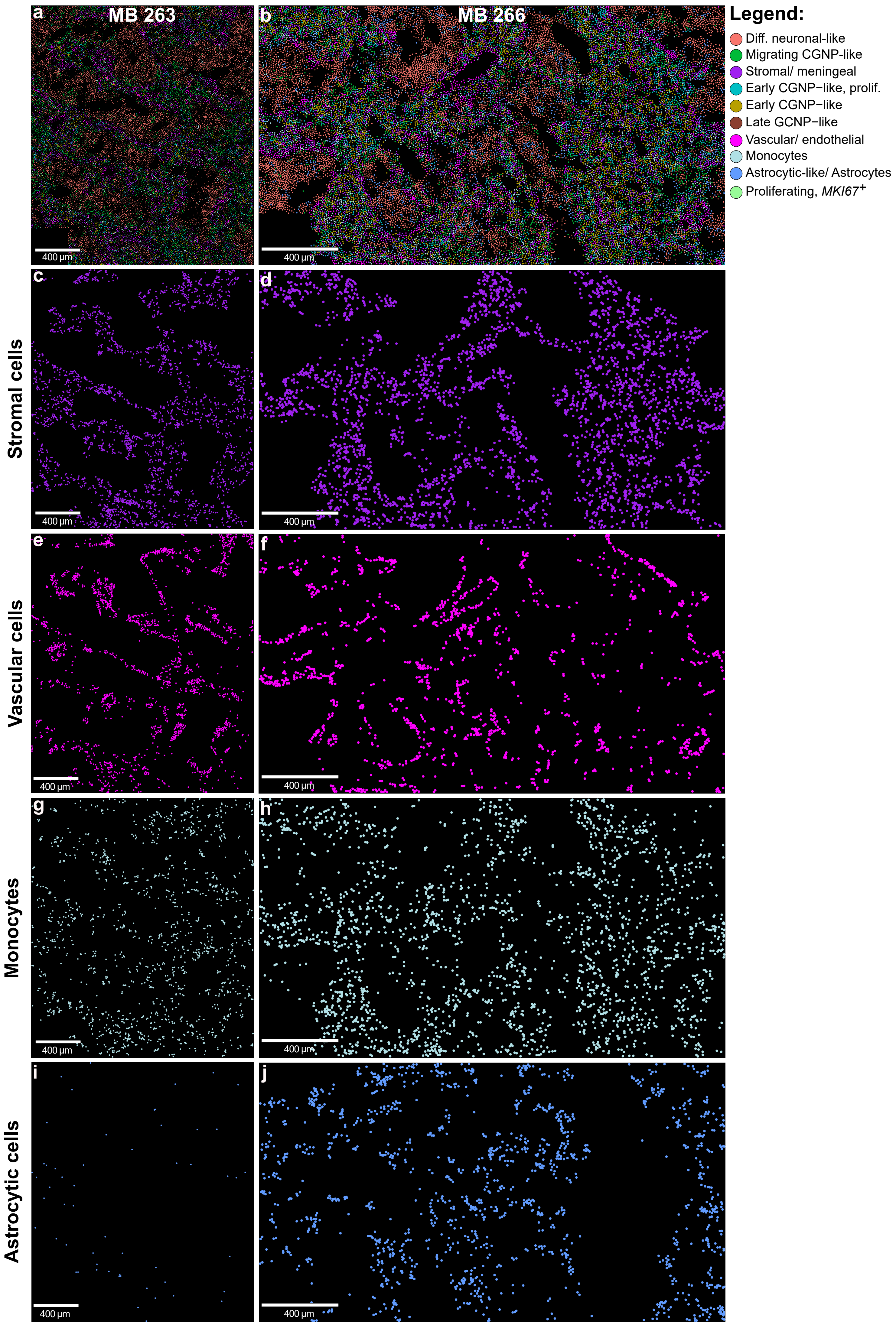

a
MB 263
b
MB 266
Legend:
Diff. neuronal-like
Migrating CGNP-like
Stromal/ meningeal
Early CGNP−like, prolif.
Early CGNP−like
Late GCNP−like
Vascular/ endothelial
Monocytes
Astrocytic-like/ Astrocytes
Proliferating, MKI67+
Stromal cells
Vascular cells
Monocytes
Astrocytic cells
c
d
e
f
g
h
i
j
400 µm

Suppl. Fig. 15: Non-malignant cells in MBEN are enriched in the internodular compartment (MB263 and MB266).

a, b Scans of MB263 (a, c, e, g, i) and MB266 (b, d, f, h, j) with mapping of single cells which recapitulate MBEN histology (**a** represents a duplication of Fig. 7f for reference). **c, d** Mapping of stromal cells. **e, f** Mapping of vascular cells. **g, h** Mapping of microglia/macrophages. **i, j** Mapping of non-malignant astrocytes and astrocytic like malignant cells. Scale bars = 400 µm.

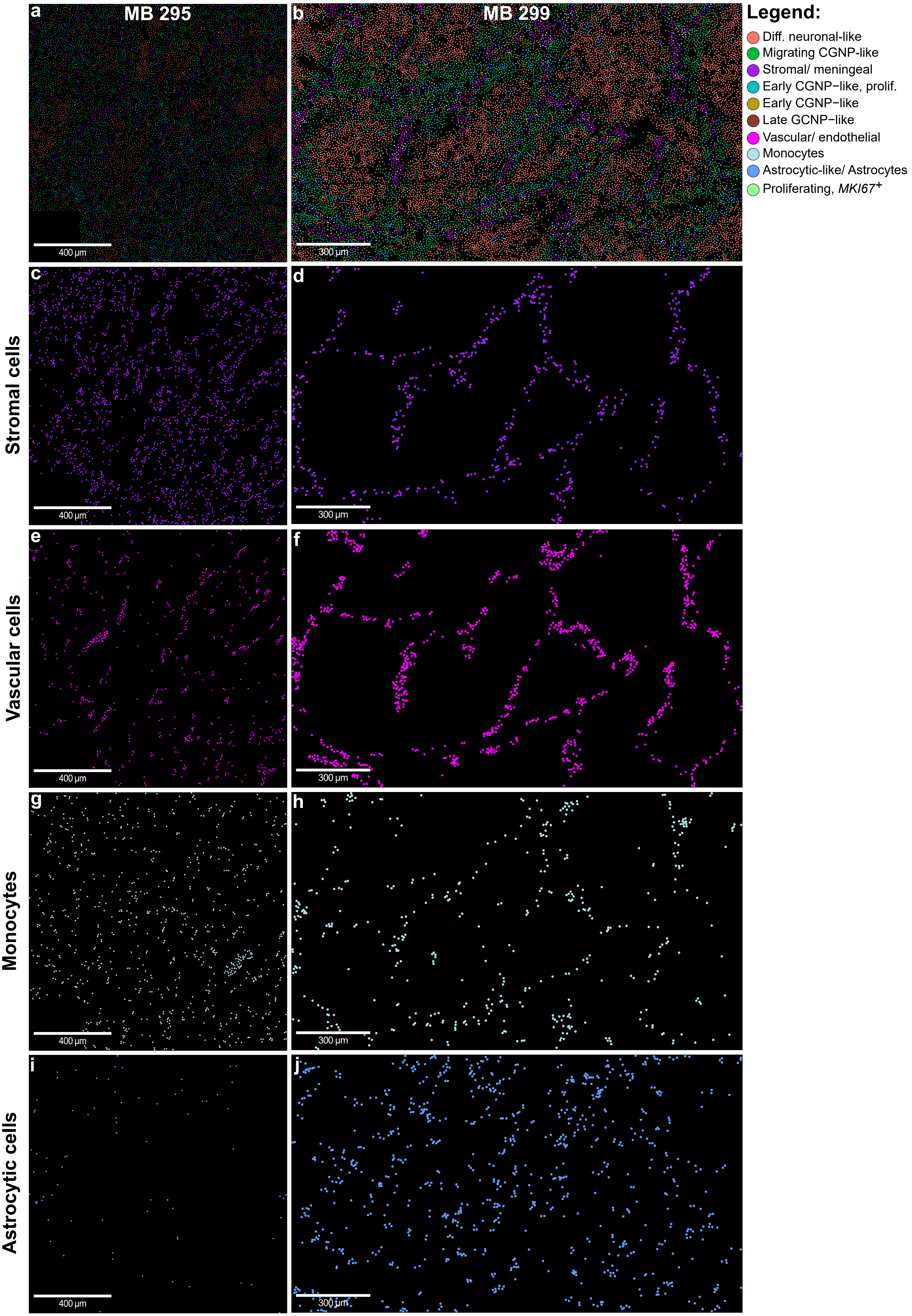
a
MB 295
b
MB 299
Legend:
Diff. neuronal-like
Migrating CGNP-like
Stromal/ meningeal
Early CGNP−like, prolif.
Early CGNP−like
Late GCNP−like
Vascular/ endothelial
Monocytes
Astrocytic-like/ Astrocytes
Proliferating, MKI67+
Stromal cells
Vascular cells
Monocytes
Astrocytic cells
c
d
e
f
g
h
i
j
400 µm
300 µm

Suppl. Fig. 16: Non-malignant cells in MBEN are enriched in the internodular compartment (MB295 and MB299.)

a, b Scans of MB295 (a, c, e, g, i) and MB299 (b, d, f, h, j) with mapping of single cells which recapitulate MBEN histology. **c, d** Mapping of stromal cells. **e, f** Mapping of vascular cells. **g, h** Mapping of microglia/macrophages. **i, j** Mapping of non-malignant astrocytes and astrocytic like malignant cells. Scale bars in a, c, e, g, i = 400 µm. Scale bars in b, d, f, h, j = 300 µm.

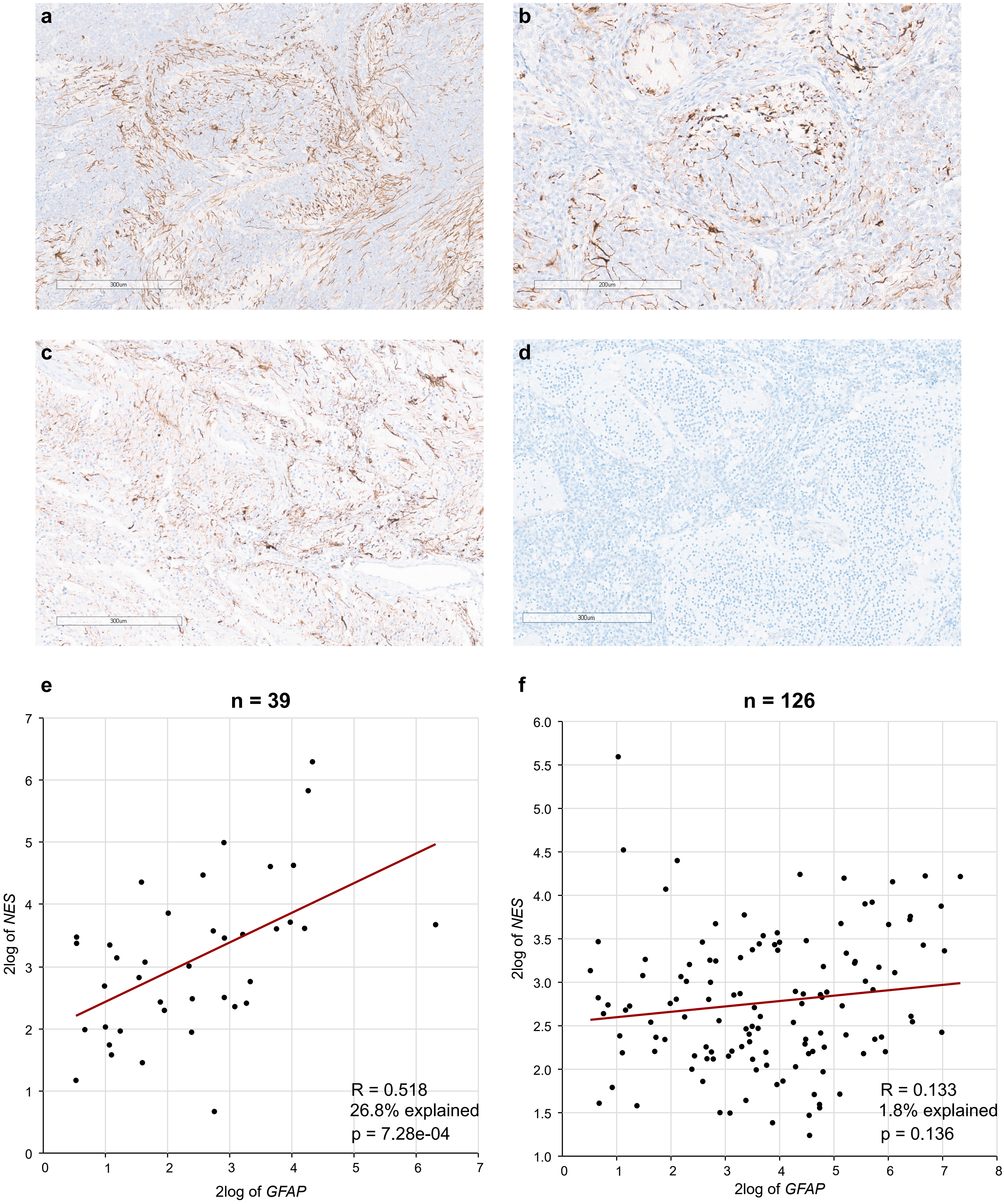

a
300um
b
200um
c
300um
d
300um
e
n = 39
2log of NES
2log of GFAP
R = 0.518
26.8% explained
p = 7.28e-04
f
n = 126
2log of NES
2log of GFAP
R = 0.133
1.8% explained
p = 0.136

Suppl. Fig. 17: Immunohistochemistry and gene expression analysis show differences in astrocytic marker expression between MBEN subtypes and DNMBs.

a, b Representative scans of GFAP-positive stainings in MBEN show expression in the peripheral regions of the nodular compartment, confirming the observations made using spatial transcriptomics (representative for n = 9/12 MBEN that expressed GFAP, also compare Suppl. Tbl. 1). **c** Expression of Nestin is similar to GFAP-expression (representative for n = 7/12 MBEN that expressed Nestin, also compare Suppl. Tbl. 1). **d** Representative example for a GFAP-negative MBEN (subtype TCL1) (representative for n = 3/12 MBEN that did not express GFAP, also compare Suppl. Tbl. 1). **e, f** The expression of *Nestin* (NES) and *GFAP* shows significant positive correlation in (e) MBEN as compared to (f) DNMB as calculated via Pearson Correlation tests. Scale bars in a, c, d = 300 µm. Scale bar in b = 200 µm. Source data are provided as a Source Data file.

Supplementary Tables

Tumor ID	TCL-subtype	GFAP	Nestin
MB274	1	No	No
201465960060_R02C01	1	No	No
201959740066_R07C01	1	Low	No
201490020210_R01C01	1	Low	No
202176290097_R04C01	1	High	High
MB266	2	High	Low
MB299	2	High	High
201490020243_R07C01	2	High	Low
201490020210_R08C01	2	High	Low
203218690035_R07C01	2	High	Low
MB263	2	No	No
201465960059_R03C01	2	High	Low

Suppl. Tbl. 1: Stainings for GFAP and Nestin in MBEN.

Immunohistochemical stainings for Nestin and GFAP show differences in expression levels between the two transcriptomic MBEN-subtypes TCL1 and TCL2.
