# Supplementary material for: Compartments in medulloblastoma with extensive nodularity are connected through differentiation along the granular precursor lineage
Source: Nat Commun. 2024 Jan 8;15:269. doi: 10.1038/s41467-023-44117-x (PMC10774372; doi:10.1038/s41467-023-44117-x)
Supplement: Supplementary file 3 — Description of Additional Supplementary Files [file 41467_2023_44117_MOESM3_ESM.pdf]

Description of Additional Supplementary Files

File Name: Supplementary Data 1
Description: Sample and methods overview.

File Name: Supplementary Data 2
Description: Differentially expressed genes in MBEN.

File Name: Supplementary Data 3
Description: Gene ontology analysis.

File Name: Supplementary Data 4
Description: Relative transcription factor activities per cluster.

File Name: Supplementary Data 5
Description: Differences in transcription factor activity between cell stages in MBEN.

File Name: Supplementary Data 6
Description: Differentially expressed genes in the internodular and nodular compartment based on microdissected tissue.

File Name: Supplementary Data 7
Description: Gene probes used for smRNA-FISH (RNAscope).

File Name: Supplementary Data 8
Description: A 100 gene panel for Molecular Cartography to investigate MBEN biology.

File Name: Supplementary Data 9
Description: Differentially expressed genes in single cell clusters derived from molecular cartography.

File Name: Supplementary Data 10
Description: Gene enrichment analysis shows differences between CD16- and CD163-positive monocytes.
