# Supplementary material for: Compartments in medulloblastoma with extensive nodularity are connected through differentiation along the granular precursor lineage
Source: Nat Commun. 2024 Jan 8;15:269. doi: 10.1038/s41467-023-44117-x (PMC10774372; doi:10.1038/s41467-023-44117-x)
Supplement: Supplementary file 14 — Reporting Summary [file 41467_2023_44117_MOESM14_ESM.pdf]

nature portfolio

Corresponding author(s): Prof. Dr. Kristian W. Pajtler

Last updated by author(s): Oct 30, 2023

Reporting Summary

Nature Portfolio wishes to improve the reproducibility of the work that we publish. This form provides structure for consistency and transparency in reporting. For further information on Nature Portfolio policies, see our Editorial Policies and the Editorial Policy Checklist.

Statistics

For all statistical analyses, confirm that the following items are present in the figure legend, table legend, main text, or Methods section.

n/a	Confirmed	
☐	☒	The exact sample size (*n*) for each experimental group/condition, given as a discrete number and unit of measurement
☐	☒	A statement on whether measurements were taken from distinct samples or whether the same sample was measured repeatedly
☐	☒	The statistical test(s) used AND whether they are one- or two-sided<br>*Only common tests should be described solely by name; describe more complex techniques in the Methods section.*
☐	☒	A description of all covariates tested
☐	☒	A description of any assumptions or corrections, such as tests of normality and adjustment for multiple comparisons
☐	☒	A full description of the statistical parameters including central tendency (e.g. means) or other basic estimates (e.g. regression coefficient) AND variation (e.g. standard deviation) or associated estimates of uncertainty (e.g. confidence intervals)
☐	☒	For null hypothesis testing, the test statistic (e.g. *F*, *t*, *r*) with confidence intervals, effect sizes, degrees of freedom and *P* value noted<br>*Give P values as exact values whenever suitable.*
☒	☐	For Bayesian analysis, information on the choice of priors and Markov chain Monte Carlo settings
☒	☐	For hierarchical and complex designs, identification of the appropriate level for tests and full reporting of outcomes
☐	☒	Estimates of effect sizes (e.g. Cohen's *d*, Pearson's *r*), indicating how they were calculated

Our web collection on statistics for biologists contains articles on many of the points above.

Software and code

Policy information about availability of computer code

Data collection	No software was used for data collection.

Data analysis

Single nucleus 10X sequencing initial data analysis (alignment,gene expression counts computation) was performed with CellRanger v3.

Single nucleus Smart-seq2 reads alignment was performed with STAR v 2.4.1d, gene expression counts were computed for each cell per sample with Subread 1.6.4 tool, and afterwards merged into a gene expression matrix via custom Python scripts within the python 2.7 environment.

Cell border detection for Resolve Bioscience spatial images was performed with CellPose 2.0.5. Gene expression counts were extracted with custom Python scripts in python3.2 environment.

Single nucleus RNA-sequencing and spatial data post analysis was performed in R 4.1 environment with the following packages: Seurat v4.0.3, DecontX v1.1.0, harmony v0.1.0, sctransform v0.3.2, slingshot 2.0.0, survival 3.2.11, ggplot2 v3.3.3, survminer 0.4.9, SingleR 1.8.1, InferCNV 1.10.1, Giotto v1.1.1., Liana 0.1.6, OmnipathR 3.7.0, DecoupleR v1.3.0, Monocle2 v2.20.0, ROGUE v1.0, MuSiC v0.9, QuPath v0.3.2. Gene ontology analysis was performed using the PANTHER classification system (Annotation version: 2021-02-01) with DEGs per cluster as input (http://www.pantherdb.org/) and visualised using the server-based REVIGO web application (http://revigo.irb.hr/). The dependencies and R environment of each used R package are provided in the respective Github repository together with the analysis source code.

Analysis source code materials for snRNA-seq and spatial data are shared via the repository:
github.com/kokonech/MBEN_snData_analysis

For manuscripts utilizing custom algorithms or software that are central to the research but not yet described in published literature, software must be made available to editors and reviewers. We strongly encourage code deposition in a community repository (e.g. GitHub). See the Nature Portfolio guidelines for submitting code & software for further information.

Data

Policy information about availability of data

All manuscripts must include a data availability statement. This statement should provide the following information, where applicable:

- Accession codes, unique identifiers, or web links for publicly available datasets
- A description of any restrictions on data availability
- For clinical datasets or third party data, please ensure that the statement adheres to our policy

The snRNA-seq and bulk-sequencing (RNA, microdissected) data have been deposited in GEO database and available under the combined accession number GSE239854. All raw images and processed data after cell segmentation from spatial transcriptomics experiments have been deposited at BioImage Archive and can be accessed under the accession numbers S-BIAD825, S-BIAD826. The DNA methylation data copy number profiles and DNA sequencing mutation results were integrated from the corresponding medulloblastoma molecular landscape study deposited at European Genome-Phenome Archive under accession number EGAS00001001953. The remaining data are available within the Article, Supplementary Information or Source Data file. Source data are provided with this paper.

Research involving human participants, their data, or biological material

Policy information about studies with human participants or human data. See also policy information about sex, gender (identity/presentation), and sexual orientation and race, ethnicity and racism.

Reporting on sex and gender

Tumor material of two male and seven female patients was used in this study. Due to the rarity of MBEN and the resulting paucity of available fresh frozen tissue, it was not possible to include more cases in order to reach a balanced sex distribution. Due to this imbalance and the overall low number of patients, no sex-based analyses were performed.

Reporting on race, ethnicity, or other socially relevant groupings

Since these samples were collected retrospectively and based on material availability, we do not have information on race or ethnicitiy for this cohort.

Population characteristics

n = 9
Diagnosis: Medulloblastoma, histological subtype: Medulloblastoma with extensive nodularity (MBEN): n = 9
Sex distribution: Two female, nine male patients
Median age at diagnosis: 2 +- 0.70 years
Relapses: 3/9 patients
Deaths: 0/9 patients

Recruitment

Patients were included retrospectively based on the availability of fresh frozen tumour material.

Ethics oversight

This study was performed after approval by the ethics committee of the Medical Faculty of Heidelberg University.

Note that full information on the approval of the study protocol must also be provided in the manuscript.

Field-specific reporting

Please select the one below that is the best fit for your research. If you are not sure, read the appropriate sections before making your selection.

☒ Life sciences ☐ Behavioural & social sciences ☐ Ecological, evolutionary & environmental sciences

For a reference copy of the document with all sections, see nature.com/documents/nr-reporting-summary-flat.pdf

Life sciences study design

All studies must disclose on these points even when the disclosure is negative.

Sample size	n = 9<br>Samples size was based on the availability of fresh frozen material of previously diagnosed MBEN tumours. Since MBEN is a rare diagnosis, it was not possible to collect a larger cohort. Since the cohort size was dependent on tissue availability, no sample size calculation was performed. To our knowledge this study, together with an accompanying study by Gold et al., is the only so far published study on intratumoral heterogeneity in MBEN.
Data exclusions	No data was excluded in this study except of individual tumour cells with low quality, which were filtered out as part of the QC process based on the number of expressed genes and the presence of duplicates.
Replication	All presented methods (DNA methylation-profiling, DNA-panel sequencing, RNA-sequencing, 10X Genomics 3'-V2 snRNAseq, SMARTseq V2 snRNAseq, RNA scope and molecular cartography) were successfully performed for at least three independent samples. All replications on independent samples were successfull. No experiments using living cells or organisms were performed.
Randomization	All analyses were performed on retrospectively collected tumour material. No analyses involving living organisms or interventions of any kind were performed, therefore randomisation was not applicable.
Blinding	All analyses were performed on retrospectively collected tumour material. No analyses involving living organisms or interventions of any kind were performed. Since our study design was explorative and did not involve a specific endpoint that would be susceptible to detection or performance bias, blinding was not necessary or applicable.

Reporting for specific materials, systems and methods

We require information from authors about some types of materials, experimental systems and methods used in many studies. Here, indicate whether each material, system or method listed is relevant to your study. If you are not sure if a list item applies to your research, read the appropriate section before selecting a response.

Materials & experimental systems

n/a	Involved in the study	
☐	☒	Antibodies
☒	☐	Eukaryotic cell lines
☒	☐	Palaeontology and archaeology
☒	☐	Animals and other organisms
☒	☐	Clinical data
☒	☐	Dual use research of concern
☒	☐	Plants

Methods

n/a	Involved in the study	
☒	☐	ChIP-seq
☒	☐	Flow cytometry
☒	☐	MRI-based neuroimaging

Antibodies

Antibodies used	Mouse monoclonal GFAP (GA5; Cell signalling), Mouse monoclonal Nestin (MAB5326; Merck / Sigma-Aldrich)
Validation	The antibody against Nestin has been validated by the manufacturer (Merck / Sigma-Aldrich) by detecting Nestin in a Western Blot Analysis on 10 µg of Huvec Lysates (Dilution: 1:1000) as well as Immunohistochemistry on formalin fixed, paraffin embedded (FFPE) tissue (1:200) and Immunocytochemistry on formaldehyde fixed cultured cells (1:200) as described and stated in the manufacturer's description (https://www.merckmillipore.com/DE/de/product/Anti-Nestin-Antibody-clone-10C2,MM_NF-MAB5326).<br><br>The antibody against GFAP has been validated by the manufacturer (Cell Signaling) by detecting GFAP in a Western Blotting Analysis (Dilution: 1:1000), Immunohistochemistry on FFPE- (1:50 - 1:200), and FF-tissue (1:400 - 1:800), Immunocytochemistry (1:400 - 1:800), and Flow Cytometry (1:400 - 1:1600) as described and stated in the manufacturer's description (https://www.cellsignal.com/products/primary-antibodies/gfap-ga5-mouse-mab/3670).
